# How online ADHD-related information affects Chinese parents’ decisions?

**DOI:** 10.1007/s12519-018-0207-x

**Published:** 2018-11-26

**Authors:** Xiao-He Yu, Le Zhong, Jia Huang

**Affiliations:** 1 Department of Pediatrics, Xiangya Hospital, Central South University, No. 87 Xiangya Road, Changsha, 410008 China; 2 Department of Pediatrics, Distinct Healthcare, Shenzhen, 518000 China

**Keywords:** Attention deficit hyperactivity disorder, Decision-making conflict, Parents

## Abstract

**Background:**

Attention deficit hyperactivity disorder (ADHD) is a major public health problem in China. Parents of children with confirmed, or suspected ADHD often face a difficult process in making decisions concerning diagnosis and treatment. The internet is a major source of information for parents. The purpose of this study is to survey Chinese parental motivation and experience in using the internet to retrieve ADHD-related information, and how well online information is associated with making decisions.

**Methods:**

Parents were recruited to fill out an online questionnaire in the health portal. A total of 404 valid questionnaires were collected.

**Results:**

A total of 47.8% of parents agree that the internet helps them to understand the potential treatment options, but 77.7% of all parents still have conflict during decision-making.

**Conclusions:**

Parents search for ADHD-related information online, but their acquisition skills need to be improved. Internet information affects their health decisions. Parents still have highly conflicting decision-making. Improving the ability of parents to obtain information on the Internet may reduce the conflict in decision-making.

**Electronic supplementary material:**

The online version of this article (10.1007/s12519-018-0207-x) contains supplementary material, which is available to authorized users.

## Introduction

Attention-deficit hyperactivity disorder (ADHD) is a major public health concern. Systematic reviews indicate that the community prevalence globally is between 2% and 7%, with an average of around 5.5% [1–3]. There are approximately 10 million children between the ages of 6 and 14 with ADHD in China. Despite the large number of children affected, there is a lack of professionals needed to diagnose and treat children in China. General pediatricians receive little–no ADHD training. There are about 300 registered child psychiatrists in China [4], which means there are only two child psychiatrists per million children aged 6–14. Developmental and behavioral pediatrics is a new subspecialty in China. The Academy Section on Developmental and Behavioral Pediatrics in China was only founded in October 2011. Many parents of children suspected of having ADHD, therefore, are not able to find a qualified physician to diagnose and treat their child.

The internet is a source of information for people concerned about mental health and developmental disabilities. People, including 96% of all parents, tend to seek out information on the incidence, treatment, or increased severity of the disability [5, 6]. Surveys have found that parents of children with ADHD and ADHD adolescents themselves have expressed similar strong preferences for the internet as the ADHD information, 49% and 51%, respectively, or from a doctor, 40% and 27%, respectively [7]. Parents of children with confirmed and suspected ADHD have been found to seek relevant and accessible information about ADHD to assist them in understanding, managing, and supporting their child and make effective treatment decisions. It has reported that as high as 94% of the internet user reported that the information they found had some impact on the health decisions they made [8–11].

Parents often grapple with decision-making conflicts or uncertainty about a course of action when caring for an ADHD child. For example, parents may be hesitant to use medicine [12]. In reviewing Chinese online ADHD forums, instances of conflicted parental decision-making are readily apparent [13]. Parents who successfully resolve their conflicts are more likely to feel satisfied with their decision and more likely to maintain the treatment resulting from that decision. Conversely, unresolved decisional conflicts may lead to delayed treatment, a lack of confidence in decisions that have been made and poor treatment continuity [14, 15]. Information is available online on Chinese websites regarding childhood ADHD, but it is what motivateses Chinese parents to seek health information online, how successful they are in obtaining information, the extent of decisional conflict the parentals experience, or how able Chinese parents are to obtain information to reduce decisional conflicts, are all unknown. The purpose of the current study is to survey Chinese parents of confirmed, or suspected, ADHD children, to explore: (1) their motivations in using the internet to retrieve ADHD-related information; (2) how extensive decisional conflicts are; (3) the extent to which online information is able to reduce decision-making conflicts regarding aspects of their child’s ADHD; and (4) sociodemographic and other factors associated with decisional conflict.

## Methods

### Design

This study is a web-based, anonymous cross-sectional survey.

### Participants

Any parent of children with confirmed, or suspected, ADHD who had obtained ADHD-related information from the internet and had used that internet-sourced information to consider a treatment decision were eligible to participate.

### Recruitment

Participants were recruited through an advertisement posted during April and May 2017 on one of the Chinese health portals (http://www.39.net), which have parent forums specifically focusing on ADHD and have the highest internet traffic on this topic. The aims of the study, and the eligibility requirements were described in the advertisement, together with the online survey developed for this study.

### Measurement

The survey was anonymous and included a total of 37 Likert scales or multiple-choice questions. Parents were surveyed about their experience and skills in using the internet. Questions about the first 4 of 5 motivations for using the internet to retrieve information regarding ADHD were included. Demographic information, such as child gender, parental age, and education levels, and information about their child’s ADHD status, such as medications and other and treatments was included.

To assess the decision-making process, a 10-item, low-literacy decision conflict scale (DCS), a validated scale developed by researchers at the Ottawa Hospital Research Institute [14], was adopted and made part of the questionnaire. This scale yields a total score and 4 subscale scores which have been found to address decision-making conflicts: informed, values clarity, support, and uncertainty. Subscale scores and total scores were calculated within a range from 0 to 100 [14]. Total score > 37.5 has been shown to indicate significantly conflicted decision-making [16].

The survey was piloted to assess the ease of survey completion and question clarity. Institutional review board approval was issued by Xiangya Hospital, Central South University.

### Analysis

Differences among study variables were tested by Student’s *t* test or ANOVA for continuous variables and Chi square test or Mann–Whitney *U* test for categories of variables. Factors influencing survey completion were assessed using a stepwise multiple linear regression model. *T* test or bivariate correlation, and multiple regression analysis were used to determine relationships between scores of decisions conflict scale and demographic factors or parents’ experience of internet use. Significance level was at the rate of *P *= 0.05. Data analysis was carried out using the SPSS software package (version 16.0, SPSS, Chicago, IL).

## Results

### Response rate

A total of 2189 eligible participants began the survey. A total of 404 (18.5%) completed it. Of the 2189 participants who began the survey, 596 completed the first 7 questions. A total of 67.8% (404/596) completed all 37 questions. In examining the differences between incomplete responders (*n *= 192) and complete responders (*n *= 404), complete responders were more likely to have had their children assessed for ADHD (30.2% vs 15.6%, *χ*^2^ = 14.55, *P *= 0.00), and were 2.34 times [odds ratio (OR) = 2.34, 95% confidence interval (CI) 1.50–3.64] more likely to complete the survey.

### Demographics and internet skills

The demographic characteristics of the parents taking the survey are shown in Table 1. As is often the case with parental surveys regarding online health information about children’s disease [17], the majority of respondents (70.8%) were women. A majority of the parents (58.7%, 237/404) rated their internet skills to be an “intermediate user”, 26.2% (106/404) as an “expert user” and 15.1% (61/404) as a “beginner”. Their rated internet skills had a medium correlation with the highest educational levels (*r*_*s*_ = 0.456, *P *= 0.000).

**Table 1:** Demographic profile of respondents

Characteristics	*n* (%)
Gender	
Female	286 (70.8)
Male	118 (29.2)
Age group (y)	
< 30	91 (22.5)
30–39	238 (58.9)
40–49	67 (16.6)
≥ 50	8 (2.0)
Marital status	
Married	375 (92.8)
Widowed	2 (0.5)
Divorced	16 (4.0)
Separated	5 (1.2)
Never married	6 (1.5)
Highest level of education completed	
Less than high school	61 (15.1)
High school	125 (30.9)
Technical college	110 (27.2)
Bachelors degree	87 (21.5)
Postgraduate degree	21 (5.3)
Employment status	
Employed	319 (79.0)
Not employed	77 (19.0)
Retired	8 (2.0)
Disabled, not able to work	0 (0)

### ADHD diagnosis and treatment details of respondents’ children

Only 30.2% of the children (122/404) had been evaluated as having ADHD by professionals. However, 82.0% (100/122) had been diagnosed. Of the 100 children diagnosed with ADHD, 17.0% had been diagnosed 6–10 years previously; 52.0% 1–6 years previously; and, 31.0% less than a year previously. Thirty parents were unsure of the type of ADHD their children had. Among the children with a confirmed ADHD type, 44.3% (31/70) were inattentive, 20% (14/70) were hyperactive–impulsive, and 35.7% (25/70) were combined.

Forty-four percent of the children with confirmed ADHD (44/100) had taken medications such as Ritalin, Concerta, or Strattera, the only three medications allowed for use in China to treat ADHD. Thirty-two were still taking medications during the survey period. There were significantly more children with confirmed ADHD who had used alternative treatments than those with unconfirmed ADHD (42.0% vs 19.4%, *χ*^2^ = 20.484, *P *= 0.000). A majority of the parents of children with either confirmed, or unconfirmed, ADHD had used parenting strategies (70.0% vs 69.7%, *χ*^2^ = 0.002, *P *= 1.000).

### Obtaining information about ADHD

Among the parents who had sought information about ADHD online, 5.9% (24/404) indicated the internet as the only information source. In addition to the internet, 28.0% (113/404) also sought information from books, 27.0% (109/404) from professionals, 21.8% (88/404) from family or friends, 17.3% (70/404) from teachers, and 17.1% (69/404) from magazines or newspapers. Only 11.6% sought information from TV or radio.

We investigated parental motives in retrieving online ADHD-related information and focused on four drives, knowledge, uncertainty, social, and self-actualization. These have been described as drives for seeking health information on the internet [8] (Table 2). The first two most common parental motives parents were “Want to get better care for their children” (self-actualization) and “Check for information about specific symptoms of ADHD” (uncertainty). About one-third (34.2%, 138/404) indicated all four drives, 29.0% (117/404) indicated three, 35.4% (143/404) indicated two, 1.0% (4/404) indicated one, and 0.5% (2/440) indicted zero.

**Table 2:** Parents’ drives for seeking ADHD related information on the internet

Variables	*n* (%)
Knowledge drive (searching for new knowledge or verifying existing knowledge**)**	280 (69.3)
Want to find out about new treatments for ADHD	193 (47.8)
Want to find the latest research about ADHD	105 (26.0)
Want to learn more about ADHD	92 (22.8)
Want to learn about other treatments beyond those that my doctor told me about	91 (22.5)
Want to be sure the advice that my doctor gave me was correct	81 (20.0)
Uncertainty drive (to reduce feelings of uncertainty and anxiety)	334 (80.2)
Check for information about specific symptoms of ADHD	285 (70.5)
Want to check for information about specific concerns	111 (27.5)
Social drive (to share information)	228 (56.4)
Want to talk to other parents of children with ADHD	189 (46.8)
Want to get information to explain their children’s ADHD to friends, relatives, teachers and colleagues	118 (29.2)
Self-actualization drive (to increase feeling of self-esteem, and to achieve the best possible health status)	361 (89.4)
Want to take better care for their children	321 (81.9)
Want to play a larger role in their children’s care related ADHD	213 (52.7)
Want to be more confident in talking to their children’s doctors about ADHD	70 (17.3)

When asked why they preferred the internet as the information source instead of other sources, 83.7% (338/404) indicated because it was convenient, 56.4% (228/404) because they could get information that would not be easy to get elsewhere, 53.0% (214/404) because it was free, 48.0% (194/404) was to find resources and products that might be helpful, 41.8% (169/404) was to read the most up-to-date information, 28.2% (114/404) to chat with professionals or ask questions online, 28.2% (114/404) because it was anonymous, and 25.2% (102/404) to get support from, or chat with, others online. The number of times parents reported using the internet for specific topics varied widely (Table 3). Nearly all (95.3%) reported going online at least once to search for information on ADHD symptoms. More than three-fourths sought information on the causes of ADHD (83.7%) or for behavior management and parenting strategies (75.7%). Only half sought information on medications (50.5%) and complementary and alternative treatments for ADHD (51.2%). Among the parents of children who had taken mediations, 84.5% (60/71) had sought information on the medications, and they searched more often that parents of children who had not taken medication (*Z *= -6.72, *P *= 0.000).

**Table 3:** Online searching times and perceived information quality on each ADHD topic

Variables	Frequency of searching				Perceived quality		
	Never	1–5 times	6–10 times	> 10 times	Good	Fair	Poor
ADHD symptoms	19 (4.7)	254 (62.9)	68 (16.8)	63 (15.6)	175 (47.1)	192 (51.6)	5 (1.3)
Causes of ADHD	66 (16.3)	237 (58.7)	47 (11.6)	54 (13.4)	145 (44.0)	171 (52.0)	13 (4.0)
Diagnosing ADHD	113 (28.0)	214 (53.0)	34 (8.4)	43 (10.6)	102 (36.4)	159 (56.8)	19 (6.8)
Associated problems	134 (33.2)	187 (46.3)	43 (10.6)	40 (9.9)	94 (35.5)	161 (60.8)	10 (3.8)
Latest research	210 (52.0)	140 (34.7)	28 (6.9)	26 (6.4)	55 (27.6)	120 (60.3)	24 (12.1)
Long term effects of ADHD	129 (31.9)	188 (46.5)	38 (9.4)	49 (12.1)	93 (38.3)	133 (54.7)	17 (7.0)
Medications	200 (49.5)	148 (36.6)	23 (5.7)	29 (7.2)	62 (31.7)	112 (57.1)	22 (11.2)
Complementary and alternative treatment	197 (48.8)	133 (32.9)	33 (8.2)	36 (8.9)	76 (36.4)	119 (56.9)	14 (6.7)
Behavior management and parenting strategies	98 (24.3)	205 (50.7)	45 (11.1)	55 (13.6)	121 (40.4)	147 (49.2)	31 (10.4)
Educational interventions	131 (32.4)	187 (46.3)	35 (8.7)	48 (11.9)	88 (35.5)	133 (53.4)	28 (11.2)

Data are represented as *n* (%). *ADHD* attention deficit hyperactivity disorder

### Information assessment and information quality

A vast majority (91.3%, 369/404) said they had assessed the reliability of ADHD-related information retrieved from the internet, but only 29.7% (120/404) always or often assessed it. Almost all of the assessment tools for online health information recommended that the recipients check the authority and currency of information [18–22]. But only 13.6% (55/404) would check who the author was and only 20.8% (84/404) would see how current the information was. Half (49.8%, 201/404) stated they assessed the information quality on how professional the appearance of the website was.

More than half of the parents rated the quality of overall information as fair (55.7%, 225/404); 42.3% (171/404) rated it as good; and, 2.0% (8/404) as poor. When rating the quality of information for each topic (Table 3), about half rated each topic as fair. There was a slightly difference between each topic. Quality of information on “symptoms of ADHD” was evaluated best, while that on “latest research about ADHD” worst.

Most of the parents (60.9%, 246/404) thought they had, or might have, found wrong or misleading information. About one-third found wrong or misleading information in the topics on ADHD diagnosis (35.0%, 86/246) and ADHD medications (30.1%, 74/246).

### Using the internet to make decisions related to ADHD

About half of parents admitted that the internet helped them to learn about available treatment options. Only about one-third knew the benefits or risks of each treatment option (Table 4). When it came to values clarity, only 30.9% were clear about which benefits mattered most to them. Only 26.7% were clear about which risks mattered most to them. Less than one-fourth felt certain enough to choose.

**Table 4:** The decisional conflicts scale

Did the use of the internet	Yes (0)	Unsure (2)	No (4)
Informed subscale, *n* (%)			
Help you know which treatment options are available to you?	193 (47.8)	174 (43.1)	37 (9.2)
Help you know the benefits of each treatment option?	134 (33.2)	212 (52.5)	58 (14.4)
Help you know the risks and side effects of each treatment option?	128 (31.7)	209 (51.7)	67 (16.6)
Values clarity subscale, *n* (%)			
Help you be clear about which benefits matter most to you?	125 (30.9)	231 (57.2)	48 (11.9)
Help you be clear about which risks and side effects matter most to you?	108 (26.7)	234 (57.9)	62 (15.3)
Support subscale, *n* (%)			
Help you get enough support from others to make a choice?	97 (24.0)	163 (40.3)	144 (35.6)
Help you make a decision without pressure from others?	158 (39.1)	141 (34.9)	105 (26.0)
Help you get enough advice to make a choice?	104 (25.7)	181 (44.8)	119 (29.5)
Uncertainty subscale, *n* (%)			
Help you be clear about the best choice for you?	121 (30.0)	212 (52.5)	71 (17.6)
Help you feel sure about what to choose?	92 (22.8)	233 (57.7)	79 (19.6)

The mean total score were as high as 44.2 ± 23.0, and 67.3% (272/404) of those parents with highly conflicted decision-making (> 37.5). The mean score of informed subscale was 37.9 ± 27.6. The values clarity subscale was 42.4 ± 28.3. The support subscale score was 50.4 ± 29.6. The uncertainty subscale was 46.1 ± 30.7. The scores of the other three subscale had correlations with score of uncertainty subscale, from high correlation to low were supported in subscale (*r*_*s*_ = 0.579, *P *= 0.000), values clarify subscale (*r*_*s*_ = 0.435, *P *= 0.000), and informed subscale (*r*_*s*_ = 0.386, *P *= 0.000).

There was a medium correlation between the score of informed subscale and score of values clarity subscale(*r*_*s*_ = 0.542, *P *= 0.000).

### Factors associating with decision conflicts

Univariate analyses showed that skill and experience with using of internet and the motives for seeking health information associated with more conflicted decision-making (Table 5). The higher the parents rated their internet skills, the more often they searched ADHD-related information. The more often parents assessed the reliability of information the higher they rated the quality of information. The more often parents could locate needed information the easier they thought it was to find what they were looking for and the less conflict they had in reaching decisions. All of the drives for seeking health information online could influence decision conflicts. Parents who had knowledge drive or social drive had lower decision conflicts; however, parents who had uncertainty drive had higher decision conflicts. For demographics factors, only education level was involved in decision conflicts. Parents who had a higher education level had lower decision conflicts. Multiple regression analysis showed that experience with using of internet had correlation with total score of DCS. The more often parents assessed the reliability of information, the better parents rated the quality of information; the more often parents could locate needed information, the lower score they had. Motives for seeking health information were linked to scores of informed subscale and support subscale. Parents with the knowledge drive thought they were better informed than parents without knowledge drive. Parents without uncertainty driver thought they were better supported for decision-making than those with uncertainty drive (Supplementary Table 1).

**Table 5:** Bivariate analysis of influence of parents’ demographics, child’s ADHD status and motives and experiences of internet using on parents’ decisional conflicts scores

Variables	Bivariate analysis				
	Informed subscale score	Values clarity subscale score	Support subscale score	Uncertainty subscale score	Total DCS score
Demographics					
Parents’ gender^a^	***t *** **= 2.15**	*t *= 0.93	*t *= 0.41	*t *= 1.93	*t *= 1.20
	***P *** **= 0.032**	*P *= 0.353	*P *= 0.683	*P *= 0.054	*P *= 0.234
Parents’ age^b^	*r*_*s*_ = − 0.04	*r*_*s*_ = − 0.01	*r*_*s*_ = 0.03	*r*_*s*_ = − 0.01	*r*_*s*_ = − 0.02
	*P *= 0.383	*P *= 0.818	*P *= 0.548	*P *= 0.875	*P *= 0.675
Education level^b^	*r*_*s*_ = − 0.10	***r***_***s***_ ** = − 0.12**	*r*_*s*_ = − 0.07	*r*_*s*_ = − 0.04	***r***_***s***_ ** = − 0.11**
	*P *= 0.053	***P *** **= 0.014**	*P *= 0.158	*P *= 0.477	***P *** **= 0.034**
Employment status^c^	*F *= 1.42	*F *= 1.21	*F *= 1.28	*F *= 0.54	*F *= 0.92
	*P *= 0.225	*P *= 0.304	*P *= 0.278	*P *= 0.704	*P *= 0.453
Marriage status^c^	*F *= 1.71	*F *= 0.43	*F *= 1.69	*F *= 2.21	*F *= 2.02
	*P *= 0.147	*P *= 0.788	*P *= 0.152	*P *= 0.068	*P *= 0.090
Child’s ADHD status					
ADHD diagnosis^a^	***t *** **= 2.15**	*t *= 1.59	*t *= 1.71	*t *= 1.54	***t *** **= 2.36**
	***P *** **= 0.013**	*P *= 0.113	*P *= 0.089	*P *= 0.124	***P *** **= 0.019**
Medication taking^a^	*t *= 1.86	*t *= 1.66	*t *= − 0.33	*t *= 0.42	*t *= 1.06
	*P *= 0.063	*P *= 0.096	*P *= 0.745	*P *= 0.677	*P *= 0.288
Using complementary and alternative medicine^a^	***t *** **= 2.50**	*t *= 1.66	*t *= 1.31	*t *= 0.49	*t *= 1.95
	***P *** **= 0.013**	*P *= 0.099	*P *= 0.190	*P *= 0.624	*P *= 0.052
Skill and experience with using the internet					
Internet skill^b^	***r***_***s***_ **= 0.10**	***r***_***s***_ **= 0**.**14**	***r***_***s***_ **= 0.12**	***r***_***s***_ **= 0.14**	***r***_***s***_ **= 0.14**
	***P *** **= 0.039**	***P *** **= 0.006**	***P *** **= 0.017**	***P *** **= 0.004**	***P *** **= 0.004**
Get information from professionals^a^	*t *= 1.08	*t *= 1.07	*t *= 1.16	*t *= 0.55	*t *= 1.25
	*P *= 0.281	*P *= 0.284	*P *= 0.245	*P *= 0.585	*P *= 0.212
Get information from books^a^	*t *= 1.08	***t *** **= 2.23**	*t *= − 0.28	*t *= − 0.78	*t *= 0.62
	*P *= 0.283	***P *** **= 0.027**	*P *= 0.780	*P *= 0.438	*P *= 0.538
Searching times^b^	***r***_***s***_ **= − 0.17**	***r***_***s***_ **= − 0.21**	***r***_***s***_ **= − 0.19**	***r***_***s***_ **= − 0.16**	***r***_***s***_ **= − 0.22**
	***P *** **= 0.001**	***P *** **= 0.000**	***P *** **= 0.000**	***P *** **= 0.001**	***P *** **= 0.000**
Information reliability assessment^b^	***r***_***s***_ **= 0.19**	***r***_***s***_ **= 0.20**	***r***_***s***_ **= 0.25**	***r***_***s***_ **= 0.24**	***r***_***s***_ **= 0.30**
	***P *** **= 0.001**	***P *** **= 0.000**	***P *** **= 0.000**	***P *** **= 0.000**	***P *** **= 0.000**
Perceived information quality^b^	***r***_***s***_ **= 0.32**	***r***_***s***_ **= *0.*18**	***r***_***s***_ **= 0.24**	***r***_***s***_ **= 0.23**	***r***_***s***_ **= 0.31**
	***P *** **= 0.000**	***P *** **= 0.000**	***P *** **= 0.000**	***P *** **= 0.000**	***P *** **= 0.000**
Locate needed information^b^	***r***_***s***_ **= 0.25**	***r***_***s***_ **= 0.14**	*r*_*s*_ = 0.06	***r***_***s***_ **= 0.11**	***r***_***s***_ **= 0.16**
	***P *** **= 0.000**	***P *** **= 0.007**	*P *= 0.198	***P *** **= 0.022**	***P *** **= 0.001**
Ease of finding information^b^	*r*_*s*_ = 0.06	*r*_*s*_ = *0.*06	*r*_*s*_ = 0.06	***r***_***s***_ **= 0.14**	***r***_***s***_ **= 0.19**
	*P *= 0.246	*P *= 0.243	*P *= 0.206	***P *** **= 0.004**	***P *** **= 0.000**
Motives for seeking health information					
Knowledge drive^a^	***t *** **= 3.84**	***t *** **= 3.16**	*t *= 1.48	*t *= 1.61	***t *** **= 3.16**
	***P *** **= 0.000**	***P *** **= 0.002**	*P *= 0.141	*P *= 0.108	***P *** **= 0.002**
Uncertainty drive^a^	*t *= − 0.98	*t *= − 0.181	***t *** **= − 2.46**	***t *** **= − 2.30**	***t *** **= − 1.96**
	*P *= 0.328	*P *= 0.856	***P *** **= 0.014**	***P *** **= 0.022**	***P *** **= 0.050**
Social drive^a^	***t *** **= 3.10**	***t *** **= 3.01**	***t *** **= 2.28**	*t *= 0.852	***t *** **= 2.97**
	***P *** **= 0.002**	***P *** **= 0.003**	***P *** **= 0.023**	*P *= 0.395	***P *** **= 0.003**
Self-actualization drive^a^	*t *= 0.60	*t *= 0.156	*t *= − 1.82	*t *= − 0.17	*t *= −0.49
	*P *= 0.547	*P *= 0.876	*P *= 0.070	*P *= 0.865	*P *= 0.624

Bold values indicate significant difference. *ADHD* attention deficit hyperactivity disorder, *DCS* decision conflict scale. ^a^Student’s *t* test, ^b^Spearman rank correlation, ^c^Mann–Whitney *U* test

## Discussion

Online surveys are not difficult to conduct, and are a cost-effective way to reach large numbers of respondents. In this study, an online survey is the most convenient way to reach parents who searched for ADHD-related information online for their children. Although, the response rates of online survey usually are low, the findings are still supposed to have validity and some kind of generalizability [23–26].

We reviewed the responses to some questions between the completed and incomplete questionnaires to find concordances and differences. Concordance of responses reflected the generalizability of our findings. Parents of children who had an ADHD assessment, and parents who searched more online were the most likely to complete the survey.

Although there are males using the internet more than females in China (55.9% vs 44.1%) [7], more mothers (70.8%) searched for online ADHD-related information than did fathers. This is consistent with survey results of parents on online health information of childhood diseases in UK [17] or US [27] and a survey conducted by a Chinese ADHD online forum. Mothers were 78.9% (341/432) of users, while only 21.1% (91/432) were fathers [28]. Mothers are the dramatically more likely to search for health information than fathers.

Among parents who suspected their children had ADHD and searched ADHD-related information online, only 30.2% had their child evaluated by professionals for ADHD. This may be partly attributable to the lack of professionals and the many difficulties surrounding clinical visits in China. ADHD treatment guidelines in China recommended medication and psychosocial treatment and warn that alternative treatments have not proved effective in treating ADHD [4]; alternative treatments are prevalent in China. A search in “China national knowledge infrastructure” (http://www.cnki.net), the world’s largest database of research content from China, retrieved 131 original articles on ADHD treatments, published between January 2006 and September 2011. Sixty-two percent (81/131) of them were on alternative treatments, such as biofeedback, herbal regimens, sensory integration therapy and others. Forty-eight percent (64/131) were on medication and only 3.1% (4/131) were on psychosocial treatments such as parenting training and behavior modification. Other than a reluctance for professional psychosocial treatment, parents showed their passion about it. Over three-quarters of parents had searched information on behavior management and parenting strategies online and nearly 70% indicated that they had used parenting strategies, such as realizing the child has unique differences and may need to be parented in a different way than usual, recognized the potential for physical and/or psychological harm to the child because of being with family members who lack understanding about ADHD, and limiting the child’s association with them; or working closely with schools.

Parents who searched ADHD-related information online did not usually retrieve information from other sources. More than half did not obtain information from professionals or books, the two additional sources they thought were useful. Although they believe in information supplied by professionals, less than one-third obtained ADHD related information from professionals. Seeing the huge amount of information disseminated on the internet, many people needed their physician to manage the information and help them reach a decision [29]. However, the majority of parents in our survey did search for information in isolation without referring to a professional. In China, most teachers lack adequate knowledge of ADHD, and they were more likely to view ADHD as either parental failure in discipline or parenting or the child’s effort than did American teachers [30], which may be why parents, in this survey, did not rate teachers as a helpful source of ADHD information.

To gain insight into people’s motives for seeking health information on the internet, Boot and Meijman systematically reviewed literature from the fields of psychology, mass communication, library and information science, and medical science, and identified five drives. They are the drive: regarding retrieving knowledge; tempering uncertainty; obtaining social contact or support; for self-actualization; and, entertainment [8]. Our survey investigated the first four drives and found that 98.5% of respondents had two or more drives. This finding confirmed our hypothesis that these drives are “interconnected and may also be present simultaneously”.

The most common drive was “self-actualization”; people search for health information to obtain the best possible health status, or in our case “to take better care for their children”. Uncertainty and fear are important factors in health communication. An important part of a general practitioner’s role is to reassure anxious patients. Now, the internet had become a significant source in addition to practitioners for parents to search for health information and to reduce uncertainty and anxiety about their child’s condition. In our survey, 80.2% of parents had indicated uncertainty.

The internet offers many opportunities, to exchange knowledge and obtain social support [31]. The internet can offer an opportunity for parents to connect with one another in common circumstances. This is not less easy outside the virtual world, because parental circumstances are not common or visible in their own communities. Nearly half of the parents surveyed wanted “to talk to other parents of children with ADHD and hear about their experience” online, but only one-quarter indicated that getting “support from or chat with others online” was an advantage of the internet. Lack of qualified online ADHD forums in China might contribute to this. It is difficult for Chinese parents to find alliances online.

Our findings suggest that convenience is a key factor driving internet use. The internet is an effective information-seeking tool. When concerns arise, a quick search will often yield plenty of information sources without the hassle of arranging a clinic visit or buying a book on the topic. The anonymity of the internet did not appeal to a majority of parents in our survey, which may be that ADHD children are thought to be naughty but smart in China, and most people are not embarrassed to have an ADHD child [30].

As parents share the decision-making process with physicians regarding the medical care their children receive, their knowledge and opinions on ADHD play a significant roles. There is an increasing need for parents of ADHD children to receive high-quality information upon which to base their decisions. Ninety-eight percent (98%) of the parents surveyed believed that the information they found was good or fair. However, this confidence may be misplaced. When assessing the quality of online ADHD-related information appearing on Chinese websites, using standard texts such as “Guidelines for the Diagnosis and Management of Attention-Deficit/Hyperactivity Disorder in Children” [4], 56% of the websites were rated as poor [32]. How did parents decide whether to trust the information they found online? Our survey indicated that they are likely to adopt different trust criteria from experts, and are more readily influenced by the appeal of the web design rather than the authority and currency of the website. Although most parents rated their internet skills as “intermediate” or “expert”, many did not have appropriate knowledge to assess the reliability of the information they retrieved from the internet. The World Wide Web is uncontrolled and unmonitored publishing. Website consumers rely more on esthetics than content. Malicious sites with “official looking” pages will mislead consumers into believing they are authoritative. Physicians are increasingly faced with the task of helping parents sift through the diverse range and quality of online health information. Their task is made more difficult when online health information appears to “scientifically valid” but is in fact not evidence-based, makes erroneous references to scientific evidence, or is laden with emotional testimonials. So parent must be made aware of potential complications-dangers from the use of web-based medical sources. Governmental websites (ending in.nhs.uk or.gov.uk) give factually correct advice and should be promoted as the first port of call for parents. The information provided by educational institution websites can be also reliable.

Parents play an active role in decision-making in managing ADHD. They decide whether to see a doctor, which treatments are performed, whether to take prescribed medications, whether to return to the clinic or discontinue treatment, and other actions so on. To make effective decisions, parents need relevant information and clarity about their values [12]. As they get information on available treatment options, and the benefits and risks of each treatment option, and clarify which benefits and risks matter most to them, they are more able to state clearly what they believe is their best choice. The majority of those polled using the internet reported that the information they found influenced the health decisions they made [9–11], 77.7% of the parents in our survey still had significant difficulties during decision-making. Parents who searched more, assessed reliability of information more, and rated information according to its quality better, may have a lower decision conflict.

Further research is required to more clearly delineate how misconceptions and inaccurate knowledge surrounding ADHD interventions affect utilization and outcomes. Simultaneously, patient training on how to utilize the network and how to identify the reliability of network information is needed. Directing patients to an approved website should improve their education and experience. In China, high-quality websites are not good enough, and more work is needed.

## Electronic supplementary material

Below is the link to the electronic supplementary material.

Supplementary material 1 (DOCX 19 kb)
