# Supplementary material for: How online ADHD-related information affects Chinese parents’ decisions?
Source: World J Pediatr. 2018 Nov 26;15(1):57–65. doi: 10.1007/s12519-018-0207-x (PMC6394639; doi:10.1007/s12519-018-0207-x)
Supplement: Supplementary file 1 — Supplementary material 1 (DOCX 19 kb) [file 12519_2018_207_MOESM1_ESM.docx]

**Supplementary Table 1.**Multivariate analysis of variablesinfluencing parents’ decisional conflicts scores

| Variables | Linear Regression multivariate analysis | | | | |  |
| --- | --- | --- | --- | --- | --- | --- |
|  | Informed subscalescore | Values clarity subscalescore | Support subscale score | Uncertainty subscale score | Total DCS score |  |
| Child’s ADHD status | |  |  |  |  |  |
| Using complementary and alternative medicinea | β= - 0.107, P=0.026 | β= - 0.022, P=0.665 | β= - 0.063, P=0.199 | β=0.000, P=0.996 | β= - 0.074, P=0.118 |  |
| Skill and experience with using the internet | | | |  |  |  |
| Searching times | β= - 0.032, P=0.541 | β= - 0.131, P=0.013 | β= - 0.103, P=0.033 | β= - 0.114, P=0.018 | β= - 0.149, P=0.001 |  |
| Information reliability assessmentb | β= 0.120, P=0.011 | β= 0.133, P=0.007 | β= 0.195, P=0.000 | β= 0.189, P=0.000 | β= 0.217, P=0.000 |  |
| Perceived information qualityc | β= 0.255, P=0.000 | β= 0.153, P=0.001 | β= 0.217, P=0.000 | β= 0.206, P=0.000 | β= 0.281, P=0.000 |  |
| Locate needed informationd | β= 0.151, P=0.002 | β= 0.054, P=0.289 | β= - 0.032, P=0.526 | β= - 0.006, P=0.911 | β= 0.063, P=0.206 |  |
| Motives for seeking health information | | | | |  |  |
| Knowledge drivee | β= - 0.105, P=0.032 | β= - 0.108, P=0.041 | β= - 0.015, P=0.784 | β= 0.031, P=0.551 | β= - 0.052, P=0.301 |  |
| Uncertainty drivee | β= 0.003, P=0.955 | β= - 0.033, P=0.517 | β= 0.114, P=0.017 | β=0.064, P=0.183 | β=0.034, P=0.461 |  |
| Social drivee | β= -0.037, P=0.446 | β= - 0.060, P=0.235 | β= - 0.080, P=0.109 | β= 0.010, P=0.834 | β= - 0.062, P=0.184 |  |

*ADHD*attention deficit hyperactivity disorder, *DCS* decision conflict scale. Value assignment:^a^0 = no; 1 = yes, ^b^1 = always; 2 = often; 3 = sometimes; 4 = seldom; 5 = never, ^c^1 = very good; 2 = good; 3 = fair; 4 = bad, ^d^1 = often; 2 = sometimes; 3 = seldom; 4 = never, ^e^0 = no drive; 1 = showing drive
